# The validity of the claims-based definition of rheumatoid arthritis evaluated in 64 hospitals in Japan

**DOI:** 10.1186/s12891-021-04259-9

**Published:** 2021-04-22

**Authors:** Kiyoshi Kubota, Masaki Yoshizawa, Satoru Takahashi, Yoshiaki Fujimura, Hiroko Nomura, Hitoshi Kohsaka

**Affiliations:** 1 NPO Drug Safety Research Unit Japan, 6-2-9-2F, Soto-Kanda, Chiyoda-ku, Tokyo, 101-0021 Japan; 2 Department of Rheumatology, Shonan Kamakura General Hospital, Kamakura, Kanagawa Japan; 3 Tokushukai General Incorporated Association Tokyo Headquarters, Chiyoda-ku, Tokyo, Japan; 4 Tokushukai Information System, Inc., Osaka-shi, Osaka, Japan; 5 Tokushukai General Incorporated Association Osaka Headquarters, Osaka-shi, Osaka, Japan; 6 Rheumatology Center, Chiba-Nishi General Hospital, Matsudo, Chiba, Japan

**Keywords:** Rheumatoid arthritis, Validation study, Sensitivity, Specificity, Positive predictive value, Negative predictive value, Bias analysis

## Abstract

**Background:**

An administrative database covering a whole population such as the national database in Japan may be used to estimate the nationwide prevalence of diseases including rheumatoid arthritis (RA) when a well-validated definition of the disease is available. In Japan, the record linkage between the administrative database and medical charts in hospitals is strictly prohibited. A “hospital-based” validation study is one of few possible validation studies where claims kept inside the study hospital are rearranged into the database structure.

**Methods:**

We selected random samples of 19,734 patients from approximately 1.6 million patients who received medical care between February 2018 and January 2019 in one of the 64 hospitals of the Tokushukai Medical Group. We excluded patients whose observation period was less than 365 days and identified 334 patients who met the definition of “possible cases of RA” whose medical charts were then independently evaluated by two rheumatologists. In a sensitivity analysis, we assessed bias due to misclassifying some patients with RA who did not meet the definition of “possible cases of RA” as a patient with no RA.

**Results:**

The kappa coefficient between the two rheumatologists was 0.80. The prevalence of RA in the study population was estimated to be 0.56%. We found that [condition code of RA] and ([any disease-modifying antirheumatic drug] or [oral corticosteroid with no systemic autoimmune diseases (other than RA) and no polymyalgia rheumatica]) had a relatively high sensitivity (approximately 73%) and a high positive predictive value (approximately 80%). In a sensitivity analysis, we found that when some patients with RA who did not meet the definition of “possible cases of RA” were misclassified as a patient with no RA, then this would lead to underestimation of the prevalence of the definition-positive patients and the adjusted prevalence.

**Conclusions:**

We recommend using the claims-based definition of RA (found in the current validation study) to estimate the prevalence of RA in Japan. We also suggest estimating the adjusted prevalence using the quantitative bias analysis method, since the prevalence of the disease in the “hospital-based” validation study is different from that in the administrative database.

**Trial registration:**

The current study is not a clinical trial and hence not subject to trial registration.

**Supplementary Information:**

The online version contains supplementary material available at 10.1186/s12891-021-04259-9.

## Background

Administrative databases have been used to estimate the prevalence of several diseases including rheumatoid arthritis (RA) [1–4]. To estimate the disease prevalence using the administrative database, the database should cover the whole population, and a well-validated definition of the disease of interest should be used.

In Japan, the prevalence of RA was estimated to be 1.7% in a study conducted in Wakayama Prefecture in 1996 [5]. In a recent study using data from the Comprehensive Survey of Living Conditions, the prevalence of RA was estimated to be 0.75% [6]. In a study using the claims database covering 1 million subjects, the prevalence of RA was estimated as 0.6 to 1.0% in the Japanese population aged ≥16 to < 75 years [7]. Nakajima et al. used data between April 2017 and March 2018 from the National Database of Health Insurance Claims and Specific Health Checkups of Japan (NDB Japan), and reported that the prevalence of patients with RA was between 0.46 and 0.88% when seven different definitions were used [8]. They recommended “Definition 3,” which was “patients ≥16 years old with 1 International Classification of Diseases, 10th revision (ICD-10) code of RA, and prescribed any disease-modifying antirheumatic drugs (DMARDs) for at least 2 out of 12 months”. However, as acknowledged by Nakajima et al. [8], the seven definitions of RA used in the study have not been validated and “Definition 3” excludes patients with RA treated by an oral corticosteroid only but not by a DMARD.

Database studies are relatively new to clinical studies in Japan and there have only been a few validation studies of several diseases [9–11]. In North America and Europe, validation studies have often been conducted by chart reviews of patients selected from the administrative databases [12–15]. However, record linkage is strictly prohibited in studies using the administrative databases; therefore, subjects selected from the administrative databases cannot be linked to the medical charts in hospitals in Japan.

A “hospital-based” validation study is one of few possible options where the claims and the information used to issue claims kept inside the study hospital are rearranged into the database structure and where claims-based definitions are evaluated by the chart review of patients in the hospital [10]. The representativeness of the “hospital-based” validation study is therefore questionable, as the population in the validation study is generally different from the population in a future study where the validated definition is used. To improve the representativeness of the validation study, the study may be conducted in a variety of hospitals so that the population in the validation study is more representative of the population covered by the administrative database.

For chronic conditions such as RA, at least two major problems exist related to the Japanese health care system in that patients can select the hospital or clinic according to their own preference [16]. First, patients who are currently receiving care for the disease of interest (e.g., RA) in a different hospital may be excluded from the study population, as the records of certain forms of medical care (e.g., drug treatment) for such patients may only be available from the claims of a different hospital. In addition, patients who receive medical care in the study hospital just once or for a short period only may also be removed from the study population, since the claims-based definition for a chronic condition often requires information collected during a specific length of time (e.g., “three condition codes over 2 years”) [15, 17, 18].

We conducted a “hospital-based” validation study in 64 hospitals located in various parts of Japan. Our aim was to find the best claims-based definition of RA used in population-based claims databases (such as NDB Japan) to estimate the prevalence of RA.

## Methods

We used electronically available claims and clinical data from the 64 hospitals of the Tokushukai Medical Group, which are routinely collected by the Tokushukai Information System (TIS) Inc. The study was carried out in accordance with the Declaration of Helsinki and approved by the Tokushukai Group Ethics Committee [19], where obtaining the informed consent from study subjects was waived for the current study, but the Committee indicated that the conduct of the study be announced through the internet [20]. Currently, Japanese hospitals may be classified as those following the diagnosis procedure combination/per-diem payment system (DPC/PDPS, simply abbreviated as DPC) [21] and as non-DPC hospitals. The 64 study hospitals of the Tokushukai Medical Group are located in 23 of Japan’s 47 prefectures; they include 47 DPC hospitals and 17 non-DPC hospitals (see Table S2 in Additional File 1). The electronically available clinical data include coded data of conditions, the drugs used in inpatient and outpatient care, and medical procedures such as lab test, surgical operations, and rehabilitation, where codes for Japanese electronic claims [22] are used. Of those codes, condition codes are mapped to the International Classification of Diseases, tenth revision (ICD-10) codes, and drug codes are mapped to the “National Health Insurance Drug Price Standard” codes [22], which can be used to group drugs. Other electronically available data include free text in electronic medical charts and the reference letter (in PDF format). However, X-ray radiographs or other images (e.g., computed tomography or magnetic resonance imaging) are not routinely collected from the 64 study hospitals or readily accessible through the network.

During the 1-year study period between February 1, 2018 and January 31, 2019, 1,590,669 patients received outpatient care (1,575,464 patients) or inpatient care (222,131 patients) in one of the 64 hospitals. A total of 13,224 of 1,590,669 patients had a condition code of RA in at least one of 12 monthly claims issued during the study period. We selected two mutually exclusive sets (Set A and Set B) from the random samples of 19,734 patients each (approximately 1.2% of the 1,590,669 patients) so that sets A and B would include approximately 160 patients (approximately 1.2% of 13,224 patients) with a condition code of RA in at least 1 monthly claim. We expected that from random samples including 160 patients with a condition code of RA, at least 100 cases of “definite” RA would be identified by the chart review, allowing for the estimation of the sensitivity of RA within ±0.1 [23]. Of the two sets of 19,734 patients each, one set (Set A) was selected for a pilot study conducted prior to the main study, which used Set B. In the pilot study, the electronic medical charts of 20 patients who met the definition of “possible cases of RA” (Table 1) in some of the 64 hospitals were reviewed by one rheumatologist (MY) (Appendix 1, Additional File 1).

**Table 1:** Criteria for definition of “possible cases of RA” subject to chart review

Criteria for definition	N of patients
1. Condition code of RA in an outpatient or inpatient claim	133
2. Diagnosis of RA found in administrative records for rehabilitation and surgical operation	24
3. Prescription of any DMARD	67
4. Rheumatoid factor positive or anti-CCP antibody positive	71
5. “RA” + a post-positional particle ^a^ in free text	89
6. “Ri-u-ma-ti” in katakana letters meaning RA in free text	247

A patient was defined as a “possible case of RA” if the patient satisfied at least 1 of 6 criteria of the definition. A patient was counted twice or more if the patient met two or more criteria and the sum of ‘N or patients’ exceeded 334

*RA* Rheumatoid arthritis, *DMARD* Disease-modifying antirheumatic drugs, *anti-CCP antibody* Anti-cyclic citrullinated peptide antibody

^a^ Japanese particles (“joshi”) are short words like “wa”, “wo”, or “ga” indicating various grammatical functions

Following the pilot study, we started the main study using 19,734 random samples of Set B. We first excluded 6712 patients whose observation period was less than 365 days out of 19,734 patients. As the research funding was limited, we did not review the medical charts of all of the remaining 13,022 patients whose observation period was 365 days or longer, but rather selected 334 patients who met the definition of “possible cases of RA” (Table 1) to classify 13,022 patients as having RA or having no RA. We assumed that the remaining 12,688 patients who did not meet the definition of “possible cases of RA” had no RA. Since the medical chart of one of the 334 possible RA patients was unavailable, the chart review was independently performed for 333 possible RA patients by two rheumatologists (MY and HK) through the network. In the chart review, we used a PDF survey form to record relevant information, including scores according to the criteria of the American College of Rheumatology (ACR) Board of Directors and the European League Against Rheumatism (EULAR) in 2010 (ACR/EULAR classification criteria) [24] (see Appendix 1, Additional File 1 for details). The agreement of the judgment by two rheumatologists was assessed using the Cohen’s kappa coefficient. Finally, two rheumatologists resolved a disagreement where the electronic medical chart could be reviewed through the network when necessary. Disagreements were settled through discussion to obtain a final judgment on whether the patient had RA, the ACR/EULAR classification score, and whether the care for RA was given in a different hospital other than the study hospital.

Although two rheumatologists classified 333 patients into three categories—(1) having RA, (2) having no RA, or (3) RA suspected—they were informed that a “RA suspected” patient would be reclassified as a patient having no RA in the final analysis; they thus requested that patients be classified into either (1) having RA or (2) having no RA whenever possible. We evaluated 32 claims-based definitions specified by combining 3 inclusion criteria and 1 exclusion criterion (as seen in Table 2) using the reference standard where patients were classified as having RA or not having RA according to the final decision agreed upon by the two rheumatologists. It should be noted that Definition 6 in Table 4 is equivalent to “Definition 3” in the study by Nakajima et al. [8].

**Table 2:** Inclusion and exclusion criteria used in claims-based definitions of RA

Description	Code	Frequency in 12 monthly claims
Inclusion criteria		
1 Condition code of RA without “suspect” flag	1A	in 1 one or more monthly claims
	1B	in 1 inpatient monthly claim or 2 or more outpatient monthly claims
2 Prescription of any DMARD	2A	in 1 or more monthly claims
	2B	in 2 or more monthly claims
3 Prescription of oral corticosteroid	3A	in 1 or more monthly claims
	3B	in 2 or more monthly claims
Exclusion criterion		
4 Systemic autoimmune diseases (other than RA) or PMR	4	in no claim

*RA* Rheumatoid arthritis, *DMARD* Disease-modifying antirheumatic drugs, *PMR* Polymyalgia rheumatica

For each of the 32 claims-based definitions of RA, patients were classified as true positive (TP), false negative (FN), false positive (FP), or true negative (TN). Four key measures of diagnostic accuracy were estimated: sensitivity (SE), specificity (SP), positive predictive value (PPV), and negative predictive value (NPV). The total number of TNs was estimated as the number of TN cases in 333 patients whose medical charts were reviewed, plus 12,688 patients who were not selected as “possible cases of RA”.

**Fig. 1:**
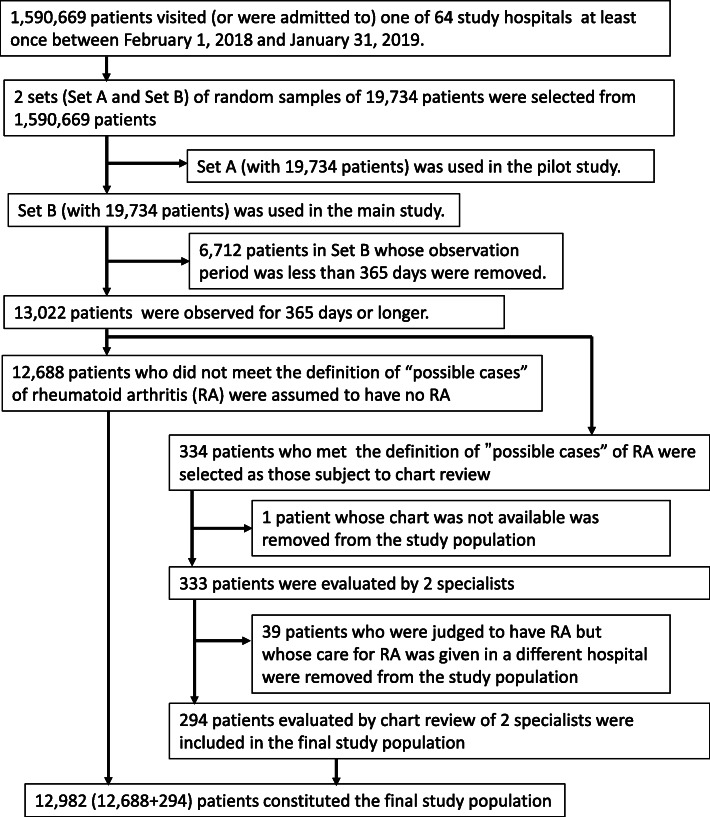
Selection of the study population and “possible cases” of RA

In the primary analysis we excluded 1 patient whose medical chart was unavailable and 39 patients receiving care for RA in a different hospital from the study population. Therefore, the size of the study population was corrected to 12,982 (Fig. 1). In the sensitivity analysis, we included 39 patients receiving care for RA in a different hospital in the study population. In the sensitivity analysis, including the 39 patients with RA, the size of the study population was 13,021 after excluding 1 patient whose medical chart was unavailable.

We also conducted another sensitivity analysis to estimate bias due to misclassification of some patients with RA as a “patient with no RA” because they did not meet the definition of “possible cases of RA” (Table 1). The sensitivity analysis was performed in conjunction with the quantitative bias analysis [25, 26], assuming that the best claims-based definition found in the current validation study would be used in future studies. We assumed that the prevalence of RA in future research would be different from the prevalence in the current validation study, but the sensitivity and specificity were the same as those in the validation study.

To calculate the adjusted number of patients with RA for future research, we used the eq. B1 = (B1*-(1-SP*)N)/(SE* + SP*-1), where B1 was the adjusted number of patients with RA, B1* was the number of definition-positive patients, N was the population size in future research where the validated definition would be used, and SE* and SP* were the sensitivity and specificity estimated in the current validation study, respectively [26]. If all patients with RA were included in 334 “possible cases of RA” in the current validation study, B1 should be the true number of patients with RA in the future research, provided that the sensitivity and specificity were the same between the validation and future studies, but if some patients with RA were not included in “possible cases of RA”, B1 would be biased.

We assumed two scenarios for future studies where the true prevalence of RA was 0.56 and 1.0%. We examined the effect of the misclassification by comparing the estimated prevalence of the definition-positive patients (B1*/N) and the adjusted prevalence (B1/N) with the true prevalence in future research (see Appendix 4 of Additional File 1 for the details). We carried out all statistical analyses using SAS 9.4 (SAS Institute, Cary, NC).

## Results

Table 3 shows the number and proportion of patients stratified by sex and age in the original population of 1,590,669 patients, subdivided by non-DPC hospitals, DPC hospitals in East Japan, and DPC hospitals in West Japan. The age-sex distribution was roughly the same between the three groups of hospitals.

**Table 3:** Age-sex distribution of the original population of 1,590,669 patients who had medical inpatient or outpatient care at least once between February 1, 2018 and January 31, 2019

	Non-DPC hospitals	DPC Hospital		Total
		East Japan	West Japan	
	17 hospitals	25 hospitals	22 hospitals	64 hospitals
Sex				
Males	69,173 (48.7%)	392,547 (49.6%)	328,752 (50.0%)	790,472 (49.7%)
Females	72,978 (51.3%)	398,158 (50.4%)	329,061 (50.0%)	800,197 (50.3%)
Age				
0–4	4904 (3.4%)	25,104 (3.2%)	37,697 (5.7%)	67,705 (4.3%)
5–14	8933 (6.3%)	42,976 (5.4%)	52,915 (8.0%)	104,824 (6.6%)
15–24	9594 (6.7%)	51,453 (6.5%)	48,714 (7.4%)	109,761 (6.9%)
25–34	11,901 (8.4%)	62,040 (7.8%)	55,298 (8.4%)	129,239 (8.1%)
35–44	16,278 (11.5%)	79,925 (10.1%)	67,202 (10.2%)	163,405 (10.3%)
45–54	17,817 (12.5%)	103,330 (13.1%)	79,469 (12.1%)	200,616 (12.6%)
55–64	18,076 (12.7%)	95,831 (12.1%)	74,005 (11.3%)	187,912 (11.8%)
65–74	22,019 (15.5%)	134,872 (17.1%)	101,639 (15.5%)	258,530 (16.3%)
75–84	18,708 (13.2%)	128,307 (16.2%)	92,824 (14.1%)	239,839 (15.1%)
85-	13,921 (9.8%)	66,867 (8.5%)	48,050 (7.3%)	128,838 (8.1%)
Total	142,151 (100.0%)	790,705 (100.0%)	657,813 (100.0%)	1590,669 (100.0%)

*DPC* Diagnosis Procedure Combination/Per-Diem Payment System

In 19,734 random samples of Set A and Set B, we found that 164 and 169 patients, respectively, had a condition code of RA on at least 1 monthly claim. After excluding 6712 patients whose observation period was less than 365 days in the main study using Set B, 133 patients had a condition code of RA in the remaining 13,022 patients. Of 133 patients, 36 patients had a condition code of RA on an inpatient claim, but 97 patients had a condition code on one or more outpatient claims only. Including those 133 patients, we selected a total of 334 patients as “possible RA patients,” and we chose 143 patients because they met only criterion 5 or 6 in Table 1 (i.e., they had the RA-related phrase in free-text in the medical chart but did not meet any other criteria).

After excluding 1 patient whose medical chart was unavailable from 334 “possible cases of RA”, 333 patients were independently evaluated by two rheumatologists, and Cohen’s kappa coefficient, used to assess the agreement of the judgment of having RA, was 0.78 when patients were classified into three categories. One rheumatologist classified 11 patients as “RA suspected,” while another rheumatologist classified 22 patients as “RA suspected.” When “RA suspected” cases were reclassified as patients having no RA, Cohen’s kappa coefficient was 0.80. After the discrepancy between the two rheumatologists was resolved through discussion, 333 possible RA patients were classified into 112 with RA, 216 with no RA, and 5 as “RA suspected.” In the final analysis, 5 “RA suspected” patients were reclassified as having no RA. Although a total of 112 patients were judged to have RA, 39 received care for RA in a different hospital; we thus removed them from the study population in the primary analysis. Among the remaining 73 patients with RA, the ACR/EULAR score was estimated as ≥6 according to the final agreement for 26 patients (36%), while in many of the remaining 47 patients with RA, the available information was insufficient to estimate the ACR/EULAR score. The prevalence of 73 patients with RA was 0.56% in the final study population of 12,982 subjects. The mean age [SD] was 73.1 [11.9] years in 62 female patients with RA, and 76.6 [9.3] years old in 11 male patients with RA. In the sensitivity analysis, we included 39 patients receiving RA care in a different hospital in the study population, and the prevalence was 0.85% (112/13,021) (Table S3, Additional File 1).

Table 4 shows the number of patients with TP, FN, FP, and TN cases as well as the estimated SE, SP, PPV, and NPV. Table 4 also displays the prevalence of definition-positive subjects. The definitions of [condition code of RA in 1 or 2 monthly claims] and [any DMARDs in 1 or 2 monthly claims] during the study period of 1 year (Definitions 5–8) had a high PPV (> 85%) but a low SE (< 60%). The definitions of [condition code of RA] and [oral corticosteroid] (Definitions 9–12) had a low PPV (< 60%) and a low SE (< 40%). The definition of [condition code of RA] and ([DMARD] or [oral corticosteroid with no systemic autoimmune diseases (other than RA) and no polymyalgia rheumatica]) (Definitions 21–28) had a relatively high SE (approximately 70%) and a high PPV (approximately 80%). The definition by [any DMARDs in 1 or 2 monthly claims] only (Definitions 3 and 4) had low SE (60–62%) but [DMARD] or ([condition code of RA in 1 or 2 monthly claims] and [oral corticosteroid with no systemic autoimmune diseases (other than RA) and no polymyalgia rheumatica]) (Definitions 29–32) had relatively high SE (75.3%) but modest PPV (approximately 70%) due to a relatively large number of FP cases.

**Table 4:** The number of patients of true positive (TP), false negative (FN), false positive (FP) and true negative (TN) and the sensitivity (SE), specificity (SP), positive predictive value (PPV) and negative predictive value (NPV), and the prevalence of the definition-positives for 32 claims-based definitions for RA

No	Definition	TP (N)	FN (N)	FP (N)	TN (N)	SE(95% CI) (%)	SP (95% CI) (%)	PPV (95% CI) (%)	NPV (95% CI) (%)	Prevalence of -positives (%)definition
1	1A	63	10	48	12,861	86.3 (78.4–94.2)	99.6 (99.5–99.7)	56.8 (47.5–66.0)	99.9 (99.9–100.0)	0.86%
2	1B	61	12	43	12,866	83.6 (75.1–92.1)	99.7 (99.6–99.8)	58.7 (49.2–68.1)	99.9 (99.9–100.0)	0.80%
3	2A	45	28	17	12,892	61.6 (50.5–72.8)	99.9 (99.8–99.9)	72.6 (61.5–83.7)	99.8 (99.7–99.9)	0.48%
4	2B	44	29	15	12,894	60.3 (49.0–71.5)	99.9 (99.8–99.9)	74.6 (63.5–85.7)	99.8 (99.7–99.9)	0.45%
5	1A and 2A	43	30	7	12,902	58.9 (47.6–70.2)	99.9 (99.9–100.0)	86.0 (76.4–95.6)	99.8 (99.7–99.9)	0.39%
6	1A and 2B	42	31	5	12,904	57.5 (46.2–68.9)	100.0 (99.9–100.0)	89.4 (80.5–98.2)	99.8 (99.7–99.8)	0.36%
7	1B and 2A	43	30	5	12,904	58.9 (47.6–70.2)	100.0 (99.9–100.0)	89.6 (80.9–98.2)	99.8 (99.7–99.9)	0.37%
8	1B and 2B	42	31	5	12,904	57.5 (46.2–68.9)	100.0 (99.9–100.0)	89.4 (80.5–98.2)	99.8 (99.7–99.8)	0.36%
9	1A and 3A	26	47	21	12,888	35.6 (24.6–46.6)	99.8 (99.8–99.9)	55.3 (41.1–69.5)	99.6 (99.5–99.7)	0.36%
10	1B and 3A	26	47	19	12,890	35.6 (24.6–46.6)	99.9 (99.8–99.9)	57.8 (43.3–72.2)	99.6 (99.5–99.7)	0.35%
11	1A and 3B	19	54	15	12,894	26.0 (16.0–36.1)	99.9 (99.8–99.9)	55.9 (39.2–72.6)	99.6 (99.5–99.7)	0.26%
12	1B and 3B	19	54	14	12,895	26.0 (16.0–36.1)	99.9 (99.8–99.9)	57.6 (40.7–74.4)	99.6 (99.5–99.7)	0.25%
13	1A and (2A or 3A)	53	20	23	12,886	72.6 (62.4–82.8)	99.8 (99.7–99.9)	69.7 (59.4–80.1)	99.8 (99.8–99.9)	0.59%
14	1A and (2B or 3A)	53	20	21	12,888	72.6 (62.4–82.8)	99.8 (99.8–99.9)	71.6 (61.3–81.9)	99.8 (99.8–99.9)	0.57%
15	1B and (2A or 3A)	53	20	19	12,890	72.6 (62.4–82.8)	99.9 (99.8–99.9)	73.6 (63.4–83.8)	99.8 (99.8–99.9)	0.55%
16	1B and (2B or 3A)	53	20	19	12,890	72.6 (62.4–82.8)	99.9 (99.8–99.9)	73.6 (63.4–83.8)	99.8 (99.8–99.9)	0.55%
17	1A and (2A or 3B)	49	24	17	12,892	67.1 (56.3–77.9)	99.9 (99.8–99.9)	74.2 (63.7–84.8)	99.8 (99.7–99.9)	0.51%
18	1A and (2B or 3B)	48	25	15	12,894	65.8 (54.9–76.6)	99.9 (99.8–99.9)	76.2 (65.7–86.7)	99.8 (99.7–99.9)	0.49%
19	1B and (2A or 3B)	49	24	14	12,895	67.1 (56.3–77.9)	99.9 (99.8–99.9)	77.8 (67.5–88.0)	99.8 (99.7–99.9)	0.49%
20	1B and (2B or 3B)	48	25	14	12,895	65.8 (54.9–76.6)	99.9 (99.8–99.9)	77.4 (67.0–87.8)	99.8 (99.7–99.9)	0.48%
21	1A and (2A or (3A and 4))	53	20	16	12,893	72.6 (62.4–82.8)	99.9 (99.8–99.9)	76.8 (66.9–86.8)	99.8 (99.8–99.9)	0.53%
22	1A and (2B or (3A and 4))	53	20	14	12,895	72.6 (62.4–82.8)	99.9 (99.8–99.9)	79.1 (69.4–88.8)	99.8 (99.8–99.9)	0.52%
23	1B and (2A or (3A and 4))	53	20	13	12,896	72.6 (62.4–82.8)	99.9 (99.8–100.0)	80.3 (70.7–89.9)	99.8 (99.8–99.9)	0.51%
24	1B and (2B or (3A and 4))	53	20	13	12,896	72.6 (62.4–82.8)	99.9 (99.8–100.0)	80.3 (70.7–89.9)	99.8 (99.8–99.9)	0.51%
25	1A and (2A or (3B and 4))	49	24	11	12,898	67.1 (56.3–77.9)	99.9 (99.9–100.0)	81.7 (71.9–91.5)	99.8 (99.7–99.9)	0.46%
26	1A and (2B or (3B and 4))	48	25	9	12,900	65.8 (54.9–76.6)	99.9 (99.9–100.0)	84.2 (74.7–93.7)	99.8 (99.7–99.9)	0.44%
27	1B and (2A or (3B and 4))	49	24	9	12,900	67.1 (56.3–77.9)	99.9 (99.9–100.0)	84.5 (75.2–93.8)	99.8 (99.7–99.9)	0.45%
28	1B and (2B or (3B and 4))	48	25	9	12,900	65.8 (54.9–76.6)	99.9 (99.9–100.0)	84.2 (74.7–93.7)	99.8 (99.7–99.9)	0.44%
29	2A or (1A and 3A and 4)	55	18	26	12,883	75.3 (65.5–85.2)	99.8 (99.7–99.9)	67.9 (57.7–78.1)	99.9 (99.8–99.9)	0.62%
30	2B or (1A and 3A and 4)	55	18	24	12,885	75.3 (65.5–85.2)	99.8 (99.7–99.9)	69.6 (59.5–79.8)	99.9 (99.8–99.9)	0.61%
31	2A or (1B and 3A and 4)	55	18	25	12,884	75.3 (65.5–85.2)	99.8 (99.7–99.9)	68.8 (58.6–78.9)	99.9 (99.8–99.9)	0.62%
32	2B or (1B and 3A and 4)	55	18	23	12,886	75.3 (65.5–85.2)	99.8 (99.7–99.9)	70.5 (60.4–80.6)	99.9 (99.8–99.9)	0.60%

The inclusion criteria (1A, 1B, 2A, 2B, 3A, 3B) and exclusion criteria (4) used in the definition are given in Table 2

For Definition 23 (or 24), where SE = 72.6% and PPV = 80.3%, for 53 TP, 20 FN, and 13 FP cases, the proportion of females was 86.8% (46/53), 80.0% (16/20), and 76.9% (10/13), respectively, and the mean age [SD] was 73.5 [11.5], 74.1 [12.1], and 69.8 [18.5] years old, respectively. In 66 definition-positive patients for Definition 23 (or 24), 84.8% (56/66) were female and the average age [SD] was 72.8 [13.1] years old. In 20 FN cases, 10 did not have a condition code of RA in any monthly claim including 7 identified as “possible cases of RA” because they met criterion 5 or 6 only (Table 1). In 13 FP cases, in addition to a condition code of RA, 4 had a condition code of systemic autoimmune diseases (3 had suspected systemic lupus erythematosus and 1 had polymyositis). Those 4 FP cases were definition-positive (for Definition 23 or 24) because they had a condition code of RA and a DMARD even if they had a condition code of systemic autoimmune diseases.

Table S3 (Additional File 1) indicates these estimates in the sensitivity study where we included 39 patients receiving care for RA in a different hospital in the study population. In Table S3, SE was 17.7 to 31.9% lower, PPV was 0.5 to 9.6% higher, and the prevalence of the definition-positive subjects was 2.6 to 15.0% higher than when we excluded 39 individuals from the primary analysis (Table 4).

**Fig. 2:**
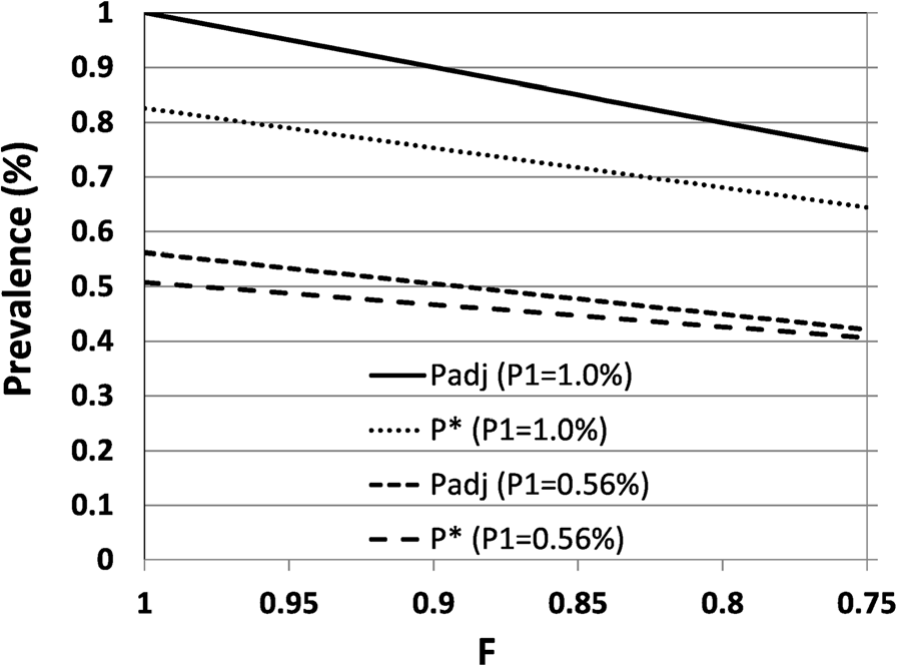
Sensitivity analysis to estimate the effect of the misclassification of patients with RA who did not meet the definition of “possible cases of RA” as those having no RA on the prevalence of definition-positive patients and the adjusted prevalence. F: the proportion of patients with RA included in “possible cases of RA”; P1: true prevalence of patients with RA in the future study to estimate the prevalence of RA; P*: the prevalence of the definition-positive patients with RA; Padj: the adjusted prevalence of the patients with RA using the method of the quantitative bias analysis; RA: rheumatoid arthritis

Figure 2 shows the results of another sensitivity analysis to estimate the effect of misclassifying patients with RA as a patient with no RA because they did not meet the definition of “possible cases of RA” (Table 1). We estimated the effect in future studies using the validated definition under 2 scenarios where the true prevalence of RA in future studies were 0.56% (as in the current validation study) or 1.0%. We assumed that in the validation study, only a fraction (F) of patients with RA were included in 333 “possible cases of RA” (after excluding 1 patient whose medical chart was unavailable from the study population); we classified the remaining patients with RA (of which the proportion was 1-F) as patients with no RA. Definition 23 in Table 4 was used to estimate the prevalence of the definition-positive subjects and the prevalence adjusted by SE* and SP* which were the sensitivity and specificity in the validation study, respectively (see Appendix 4 in Additional File 1 for the details).

When F = 1 (i.e., we included all patients with RA in “possible cases of RA”), the prevalence of the definition-positive subjects (P*) was 0.51% and the adjusted prevalence (P_adj_) was 0.56% when the true prevalence was 0.56%. Similarly, when F = 1, the definition-positive subjects (P*) was 0.83%, while the adjusted prevalence (P_adj_) was 1.0% when the true prevalence was 1.0%. When F < 1, both P* and P_adj_ were underestimated. When F < 1, the estimated values of P* and P_adj_ were approximately F times the corresponding values when F = 1.

## Discussion

In the current validation study of claims-based definitions for identifying RA, we randomly selected 19,734 patients from 1,590,669 patients who had outpatient or inpatient care in one of 64 hospitals located in 23 of Japan’s 47 prefectures. After excluding 6712 patients who were observed for less than 365 days and 39 patients receiving care for RA in a different hospital, 73 patients had RA; we estimated the prevalence of RA to be 0.56% in this population. Although our main objective was to assess the validity of the claims-based definition of RA, rather than to determine the prevalence itself, this prevalence (0.56%) was similar to that in the previous studies [5–8]. In the current validation study, we estimated four key measures of diagnostic accuracy (SE, SP, PPV, and NPV), while according to a systematic review of validation studies to identify rheumatic diseases published in 2013, authors found all of those four key measures only in 4 out of 23 studies [27]. In this systematic review, the values of those measures varied according to different sampling population sources, the sources of data for case definition, and reference standard definitions [27].

Of 32 claims-based definitions estimated in the current validation study, we found that the definition of [condition code of RA in 1 or 2 monthly claims] and [any DMARDs in 1 or 2 monthly claims] during the study period of 1 year (Definitions 5–8), including Definition 6 which is equivalent to “Definition 3” in the recent study by Nakajima et al. [8] had a high PPV (> 85%) but a low SE (< 60%). These definitions may be useful when patients without RA should be excluded as much as possible as in research where the effectiveness or safety of a drug and other interventions is determined by comparing the incidence of an outcome between the exposed and unexposed patients (or between those who had Drug A and Drug B). However, in order to measure the prevalence of RA, Definition 23 (or 24) is recommended, as it has a relatively high sensitivity (approximately 73%) and a high PPV (approximately 80%). We also suggest conducting an additional quantitative bias analysis to estimate the adjusted prevalence, since bias due to the difference in the population in the “hospital-based” validation study and populations in future research could be mitigated to some extent.

In the primary analysis, we excluded 39 patients with RA receiving care for RA in a different hospital. In the Japanese health care system, the precise definition of the population covered by one hospital is difficult [16]. As in the definition of “secondary bases” in a case-control study [28], the study population in the current validation study would be defined as “all people who would receive care for RA and be observed for 365 days or longer in the study hospital if they had RA.” We excluded 6712 patients whose observation period was less than 365 days, as well as 39 patients who had RA care in a different hospital as they were not thought to be included in the study population. Indeed, 39 patients who had RA care in a different hospital differed considerably from the 73 patients with RA who received care in the study hospital. For example, in 73 patients with RA treated in the study hospital, 63 (86.3%) had a condition code of RA in a claim, and 43 (58.9%) patients had a DMARD, while among 39 patients receiving RA care in a different hospital, only 17 (43.6%) had a condition code of RA, and only 4 (10.2%) had a DMARD. When we included the 39 patients with RA, the sensitivity and PPV were considerably different from those in the primary analysis (Table S3 in Additional File 1). We believe that excluding those 39 patients was a proper strategy to evaluate the valid claims-based definitions.

Figure 2 shows the outcomes of another sensitivity analysis, assuming that some of patients with RA did not meet the definition of “possible cases of RA.” However, it is unlikely that many patients with RA receiving care for RA in the study population did not meet any criterion of “possible cases of RA” (Table 1), including RA-related phrases in the free text of the medical chart. The electronic medical chart was used in all of 64 hospitals in the Tokushukai Medical Group and the search for RA-related phrases was almost perfect (except for handwritten phrases in the reference letter in PDF format).

A strength of the current study is that we used data from 64 hospitals located in various parts of Japan; thus, the results are likely more representative of the general population compared to studies covering only one or a few hospitals [9–11]. As shown in Table 3, the age─sex distribution was similar between small non-DPC hospitals and DPC hospitals located in East and West Japan. The boundary between primary care and second/tertiary care is indistinct in the Japanese health care system [16], and both small and large hospitals maintain large outpatient departments and provide outpatient care to nearby residents. For example, 86% out of the 1,590,669 patients received outpatient care only during the 1-year study period. The similarity of the age─sex distribution between small and large hospitals in East and West Japan reflects the fact that outpatient care in Japanese hospitals is usually open to all nearby residents, although this does not necessarily mean that the population in the current validation study is representative of Japan’s people. Another strength of the current study was that two rheumatologists were able to review the original electronic medical charts in the various hospitals through the network.

However, the current study has several limitations. Since record-linkage access in the study using the administrative database is strictly prohibited, we could not perform chart review for random samples directly selected from the administrative database. Thus, the population in the validation study is likely to differ from populations in future research where the validated definition is used. Nevertheless, the adjusted prevalence, estimated by the method of the quantitative bias analysis, will to some extent reduce bias because of the difference of the population in the validation study compared to future research, provided that the sensitivity and specificity are the same between the two studies. Another limitation was that we excluded as many as 39 patients receiving RA care in a different hospital from the main analysis, leading to reduced statistical power, although the confidence interval of SE obtained in the primary analysis (e.g., 0.73 (0.62–0.83) for Definition 23) roughly met the prespecified level (within ±0.1). Last, X-ray radiographs were not available through the network when the two rheumatologists reviewed the medical charts in the current study, which may have reduced the accuracy of the judgment on whether a patient had RA.

## Conclusion

We conducted a validation study of the claims-based definition of RA in approximately 1.6 million patients who had inpatient or outpatient care in 64 hospitals located in various areas of Japan. We found a suitable claims-based definition that may be used to estimate the prevalence of RA in future research using a population-based claims data such as NDB Japan. We recommend that (in future research to estimate the prevalence of RA) the best claims-based definition found in the current validation study (Definition 23 or 24) be used. We also suggest estimating the adjusted prevalence using the quantitative bias analysis method because the population in the “hospital-based” validation study is different from that in future research.

## Supplementary Information

**Additional file 1 Appendix 1** Pilot study and PDF survey form**. Appendix 2** Table S2: Original population and random samples selected from the original population who had inpatient or outpatient medical care in the study hospital at least once between February 1, 2018 and January 31, 2019. **Appendix 3** Table S3: The number of patients of true positive (TP), false negative (FN), false positive (FP), and true negative (TN) and the sensitivity (SE), specificity (SP), positive predictive value (PPV) and negative predictive value (NPV), and the prevalence of the definition-positive patients for 32 claims-based definitions for RA when 39 patients with rheumatoid arthritis (RA) treated in a different hospital were included in the study population. **Appendix 4**: Sensitivity analysis to evaluate the effect of potential misclassification of patients with rheumatoid arthritis (RA) who did not meet “Definitions of possible cases of RA” (Table 1) as patients having no RA on the prevalence of definition–positive patients and the adjusted prevalence.

## Data Availability

The datasets used and/or analysed during the current study are available from the corresponding author on reasonable request.

## Abbreviations

ACR/EULAR: American College of Rheumatology Board of Directors and the European League Against Rheumatism
anti-CCP: Anti-cyclic citrullinated peptide
DMARD: Disease-modifying antirheumatic drug
DPC: Diagnosis Procedure Combination/Per-Diem Payment System
ICD-10: International Classification of Diseases, 10th revision
NDB Japan: The National Database of Health Insurance Claims and Specific Health Checkups of Japan
PMR: Polymyalgia rheumatica
RA: Rheumatoid arthritis
TIS: Tokushukai Information System
FN: False negative
FP: False positive
TN: True negative
TP: True positive
SE: Sensitivity
SP: Specificity
PPV: Positive predictive value
NPV: Negative predictive value
