# Supplementary material for: The validity of the claims-based definition of rheumatoid arthritis evaluated in 64 hospitals in Japan
Source: BMC Musculoskelet Disord. 2021 Apr 22;22:373. doi: 10.1186/s12891-021-04259-9 (PMC8063301; doi:10.1186/s12891-021-04259-9)
Supplement: Supplementary file 1 — Additional file 1 Appendix 1 Pilot study and PDF survey form. Appendix 2 Table S2: Original population and random samples selected from the original population who had inpatient or outpatient medical care in the study hospital at least once between February 1, 2018 and January 31, 2019. Appendix 3 Table S3: The number of patients of true positive (TP), false negative (FN), false positive (FP), and true negative (TN) and the sensitivity (SE), specificity (SP), positive predictive value (PPV) and negative predictive value (NPV), and the prevalence of the definition-positive patients for 32 claims-based definitions for RA when 39 patients with rheumatoid arthritis (RA) treated in a different hospital were included in the study population. Appendix 4: Sensitivity analysis to evaluate the effect of potential misclassification of patients with rheumatoid arthritis (RA) who did not meet “Definitions of possible cases of RA” (Table 1) as patients having no RA on the prevalence of definition–positive patients and the adjusted prevalence. [file 12891_2021_4259_MOESM1_ESM.docx]

**The validity of the claims-based definition of rheumatoid arthritis evaluated in 64 hospitals in Japan**

Kiyoshi Kubota ^1^

Masaki Yoshizawa ^2^

Satoru Takahashi ^3^

Yoshiaki Fujimura ^4^

Hiroko Nomura ^5^

Hitoshi Kohsaka ^6^

1 NPO Drug Safety Research Unit Japan, Chiyoda-ku, Tokyo, Japan

2 Department of Rheumatology, Shonan Kamakura General Hospital, Kamakura, Kanagawa, Japan

3 Tokushukai General Incorporated Association Tokyo Headquarters, Chiyoda-ku, Tokyo, Japan

4 Tokushukai Information System, Inc., Osaka-shi, Osaka, Japan

5 Tokushukai General Incorporated Association Osaka Headquarters, Osaka-shi, Osaka, Japan

6 Rheumatology Center, Chiba-Nishi General Hospital, Matsudo, Chiba, Japan

**Additional File 1**

**Appendix 1** Pilot study and PDF survey form

**Appendix 2** Table S2: Original population and random samples selected from the original population who had inpatient or outpatient medical care in the study hospital at least once between February 1, 2018 and January 31, 2019

**Appendix 3** Table S3: The number of patients of true positive (TP), false negative (FN), false positive (FP), and true negative (TN) and the sensitivity (SE), specificity (SP), positive predictive value (PPV) and negative predictive value (NPV), and the prevalence of the definition-positive patients for 26 claims-based definitions for RA when 39 patients with rheumatoid arthritis (RA) treated in a different hospital were included in the study population

**Appendix 4**: Sensitivity analysis to evaluate the effect of potential misclassification of patients with rheumatoid arthritis (RA) who did not meet “Definitions of possible cases of RA” (Table 1) as patients having no RA on the prevalence of definition–positive patients and the adjusted prevalence

**Appendix 1**

Pilot study and PDF survey form

The main purposes of the pilot study were to confirm the practicability of the chart review through the network for patients in hospitals located in various areas in Japan and to know whether a PDF survey form used in the study could effectively collect the relevant information.

The pilot study was conducted using Set A. Of 19,734 patients in Set A, we found 469 patients who met the criteria of "possible cases of RA" (Table 1) disregarding whether or not the observation period was less than 365 days. Of 469 patients, we selected 20 patients from 16 hospitals. Of 20 patients, 10 were selected as they had the condition code of RA in the claims. Of the remaining 10 patients who did not have the condition code of RA in claims, we selected 2 patients prescribed a DMARD, 1 patient with diagnosis of RA in the list of surgical operation maintained in the hospital, 3 who had a RA-related phrase in the free text only, and 4 had positive rheumatoid factor or positive anti-CCP antibody with or without RA-related phrase in free text.

A PDF survey form was designed to record age and sex of a possible RA patient, information needed to classify patients according to the criteria by the American College of Rheumatology (ACR) Board of Directors and the European League Against Rheumatism (EULAR) in 2010 (number of swollen or tender joints, duration of symptoms, serology, and acute phase reactants), and the final judgment made by the rheumatologist about whether the patient had RA. Information on whether the patient was receiving care for RA in a different hospital was also collected by the survey form, provided that the patient was judged to have RA. We used a PDF form as the information could be transformed into a csv file without the need of manual re-entry of data.

Although the original plan was to conduct the second or third pilot study based on the results of the first pilot study if needed, we confirmed that the chart review through the network was practical and the survey form was able to collect the information effectively in the first pilot study. Thus, the main study was started after the first pilot study on 20 patients.

**Appendix 2**

Table S2: Original population and random samples selected from the original population who had inpatient or outpatient medical care in the study hospital at least once between February 1, 2018 and January 31, 2019

_______________________________________________________________________________

Hospital Prefecture Class Original Population Random Samples _

ID Total Male 　 Age Total Male 　 Age

　 N % Mean (SD) N % Mean (SD)

_______________________________________________________________________________

1 Hokkaido DPC 36,612 46.1 49.7 (26.7) 481 44.5 50.3 (26.3)

2 Hokkaido DPC 34,363 49.3 52.8 (24.7) 424 45.5 53.9 (25.1)

3 Hokkaido DPC 47,738 52.6 52.3 (23.6) 606 52.1 53.8 (24.4)

4 Hokkaido Non-DPC 8,171 51.4 46.3 (28.0) 94 47.9 41.7 (29.7)

5 Hokkaido Non-DPC 4,871 47.2 58.2 (22.1) 56 58.9 53.8 (21.5)

6 Hokkaido DPC 16,452 42.1 49.5 (26.3) 204 42.2 48.8 (25.7)

7 Hokkaido DPC 10,920 50.5 53.8 (23.5) 129 49.6 55.8 (23.1)

8 Miyagi DPC 22,898 50.4 56.9 (23.4) 297 48.5 57.6 (23.2)

9 Yamagata DPC 12,742 46.8 62.8 (20.8) 163 50.3 61.1 (20.9)

10 Yamagata Non-DPC 8,776 47.9 60.4 (22.0) 105 51.4 61.3 (21.9)

11 Yamagata Non-DPC 11,648 56.2 54.9 (21.9) 140 51.4 55.9 (21.5)

12 Ibaraki DPC 26,347 49.4 49.0 (25.1) 313 50.8 47.0 (25.8)

13 Saitama DPC 10,479 50.1 55.4 (25.0) 125 49.6 54.4 (25.0)

14 Chiba DPC 37,456 49.3 60.4 (21.1) 469 51.8 59.0 (21.3)

15 Chiba DPC 90,763 51.0 50.2 (27.2) 1,152 51.7 49.9 (27.4)

16 Chiba Non-DPC 8,106 49.6 64.4 (20.9) 99 47.5 61.9 (20.9)

17 Chiba DPC 47,048 50.7 52.1 (24.7) 595 50.8 51.1 (26.0)

18 Chiba DPC 14,445 53.2 54.5 (25.6) 180 46.7 52.6 (26.8)

19 Chiba DPC 40,886 50.0 55.7 (24.8) 494 49.6 56.5 (24.9)

20 Chiba DPC 19,974 52.6 51.9 (24.5) 249 49.0 52.3 (25.3)

21 Tokyo Non-DPC 19,782 48.0 47.8 (27.4) 240 45.4 47.4 (27.5)

22 Kanagawa DPC 63,365 48.2 54.9 (24.7) 796 47.6 53.8 (25.4)

23 Kanagawa DPC 16,956 50.4 54.4 (23.9) 197 44.2 55.4 (24.5)

24 Kanagawa DPC 93,607 46.3 57.0 (24.3) 1,198 44.4 58.7 (24.6)

25 Kanagawa DPC 6,396 53.5 69.5 (17.4) 81 50.6 70.4 (19.7)

26 Kanagawa DPC 30,648 52.4 52.8 (21.8) 364 51.4 53.6 (21.2)

27 Kanagawa Non-DPC 19,583 43.7 51.0 (25.8) 231 48.9 52.8 (26.7)

Table S2 -- continued

28 Niigata Non-DPC 3,244 44.0 65.3 (22.4) 42 54.8 50.5 (18.6)

29 Yamanashi DPC 10,898 53.0 56.2 (22.0) 134 57.5 56.1 (23.5)

30 Gifu DPC 25,932 47.3 55.5 (19.8) 309 51.8 56.0 (18.9)

31 Shizuoka DPC 15,414 51.6 57.0 (22.5) 186 55.4 56.0 (21.1)

32 Shizuoka DPC 21,083 49.8 52.5 (26.2) 264 47.0 54.3 (26.8)

33 Aichi DPC 37,283 52.8 57.3 (22.5) 474 47.5 57.4 (21.9)

34 Shiga DPC 29,643 54.0 45.9 (24.1) 343 53.4 46.0 (23.6)

35 Osaka DPC 41,064 49.5 52.3 (24.2) 495 50.3 52.5 (24.1)

36 Osaka DPC 36,308 52.9 53.3 (23.1) 432 55.1 53.1 (22.8)

37 Osaka DPC 57,211 52.3 52.5 (26.8) 681 53.2 54.0 (26.5)

38 Osaka DPC 50,219 49.5 57.1 (24.0) 639 48.7 58.0 (23.4)

39 Osaka DPC 62,831 49.0 48.6 (26.6) 785 52.2 48.5 (26.8)

40 Osaka Non-DPC 3,235 69.1 49.1 (18.6) 39 79.5 58.4 (23.6)

41 Osaka DPC 39,497 44.5 51.3 (26.7) 481 42.6 52.1 (26.6)

42 Osaka DPC 30,402 46.2 50.7 (25.6) 388 43.6 52.2 (25.3)

43 Hyogo DPC 15,612 47.0 51.0 (26.2) 189 43.9 49.0 (27.2)

44 Hyogo DPC 16,451 48.2 50.3 (26.1) 201 46.8 51.5 (27.2)

45 Nara Non-DPC 17,259 43.2 53.8 (24.7) 214 43.9 56.6 (23.5)

46 Shimane DPC 11,634 57.3 53.0 (22.0) 140 54.3 54.3 (23.3)

47 Ehime DPC 7,471 52.8 65.0 (20.6) 98 51.0 62.8 (21.2)

48 Fukuoka DPC 76,852 50.2 40.0 (28.8) 961 47.9 40.9 (29.6)

49 Fukuoka Non-DPC 3,131 47.2 51.7 (21.6) 36 47.2 58.0 (21.0)

50 Nagasaki DPC 9,991 52.6 51.5 (21.7) 121 53.7 53.3 (20.2)

51 Kagoshima DPC 11,827 46.9 47.8 (28.9) 152 49.3 48.4 (28.2)

52 Kagoshima DPC 8,930 54.1 53.9 (22.5) 113 54.9 51.8 (23.7)

53 Kagoshima DPC 19,176 50.9 62.9 (21.5) 260 45.4 62.2 (21.4)

54 Kagoshima Non-DPC 6,787 47.5 49.4 (27.9) 84 40.5 43.6 (30.3)

55 Kagoshima Non-DPC 6,047 47.6 53.8 (26.9) 72 52.8 51.2 (25.4)

56 Kagoshima DPC 8,969 47.9 50.2 (26.9) 111 48.6 48.4 (26.4)

57 Kagoshima DPC 12,739 44.1 53.8 (24.2) 158 39.2 53.9 (24.6)

58 Kagoshima Non-DPC 4,313 49.5 52.2 (27.6) 52 51.9 51.1 (28.1)

59 Kagoshima Non-DPC 2,125 46.4 58.6 (25.5) 27 44.4 65.7 (20.8)

60 Okinawa DPC 39,409 52.0 49.1 (25.5) 496 51.0 49.7 (25.8)

61 Okinawa DPC 61,694 49.3 45.1 (27.4) 779 50.2 43.8 (27.1)

Table S2 --- continued

62 Okinawa Non-DPC 4,136 48.5 50.1 (27.2) 50 56.0 45.4 (25.5)

63 Okinawa DPC 9,883 54.1 54.2 (21.3) 118 50.8 55.4 (21.2)

64 Okinawa Non-DPC 10,937 55.2 48.5 (19.9) 128 55.5 46.7 (20.4)

Total 1,590,669 49.7 52.3 (25.4) 19,734 49.2 52.5 (25.6)

_______________________________________________________________________________

DPC: Diagnostic Procedures Combination,

**Appendix 3**

Table S3: The number true positive (TP), false negative (FN), false positive (FP), and true negative (TN) patients as well as the sensitivity (SE), specificity (SP), positive predictive value (PPV), negative predictive value (NPV), and the prevalence of the definition-positive patients for 32 claims-based definitions for RA when 39 patients with rheumatoid arthritis (RA) treated in a different hospital were included in the study population in the sensitivity analysis

____________________________________________________________________________________________________________________________

No Definition TP FN FP TN SE 　 SP PPV NPV Prevalence

(N) (N) (N) (N) (95% CI) (%) (95% CI) (%) (95% CI) (%) (95% CI) (%) of definition

-positives (%)

____________________________________________________________________________________________________________________________

1 1A 79 33 48 12861 70.5 (62.1-79.0) 99.6 (99.5-99.7) 62.2 (53.8-70.6) 99.7 (99.7-99.8) 0.98%

2 1B 77 35 43 12866 68.8 (60.2-77.3) 99.7 (99.6-99.8) 64.2 (55.6-72.7) 99.7 (99.6-99.8) 0.92%

3 2A 49 63 17 12892 43.8 (34.6-52.9) 99.9 (99.8-99.9) 74.2 (63.7-84.8) 99.5 (99.4-99.6) 0.51%

4 2B 46 66 15 12894 41.1 (32.0-50.2) 99.9 (99.8-99.9) 75.4 (64.6-86.2) 99.5 (99.4-99.6) 0.47%

5 1A and 2A 47 65 7 12902 42.0 (32.8-51.1) 99.9 (99.9-100.0) 87.0 (78.1-96.0) 99.5 (99.4-99.6) 0.41%

6 1A and 2B 44 68 5 12904 39.3 (30.2-48.3) 100.0 (99.9-100.0) 89.8 (81.3-98.3) 99.5 (99.4-99.6) 0.38%

7 1B and 2A 47 65 5 12904 42.0 (32.8-51.1) 100.0 (99.9-100.0) 90.4 (82.4-98.4) 99.5 (99.4-99.6) 0.40%

8 1B and 2B 44 68 5 12904 39.3 (30.2-48.3) 100.0 (99.9-100.0) 89.8 (81.3-98.3) 99.5 (99.4-99.6) 0.38%

9 1A and 3A 30 82 21 12888 26.8 (18.6-35.0) 99.8 (99.8-99.9) 58.8 (45.3-72.3) 99.4 (99.2-99.5) 0.39%

10 1B and 3A 30 82 19 12890 26.8 (18.6-35.0) 99.9 (99.8-99.9) 61.2 (47.6-74.9) 99.4 (99.2-99.5) 0.38%

11 1A and 3B 20 92 15 12894 17.9 (10.8-25.0) 99.9 (99.8-99.9) 57.1 (40.7-73.5) 99.3 (99.1-99.4) 0.27%

12 1B and 3B 20 92 14 12895 17.9 (10.8-25.0) 99.9 (99.8-99.9) 58.8 (42.3-75.4) 99.3 (99.1-99.4) 0.26%

13 1A and (2A or 3A) 60 52 23 12886 53.6 (44.3-62.8) 99.8 (99.7-99.9) 72.3 (62.7-81.9) 99.6 (99.5-99.7) 0.64%

14 1A and (2B or 3A) 59 53 21 12888 52.7 (43.4-61.9) 99.8 (99.8-99.9) 73.8 (64.1-83.4) 99.6 (99.5-99.7) 0.61%

Table S3 -- continued

15 1B and (2A or 3A) 60 52 19 12890 53.6 (44.3-62.8) 99.9 (99.8-99.9) 75.9 (66.5-85.4) 99.6 (99.5-99.7) 0.61%

16 1B and (2B or 3A) 59 53 19 12890 52.7 (43.4-61.9) 99.9 (99.8-99.9) 75.6 (66.1-85.2) 99.6 (99.5-99.7) 0.60%

17 1A and (2A or 3B) 54 58 17 12892 48.2 (39.0-57.5) 99.9 (99.8-99.9) 76.1 (66.1-86.0) 99.6 (99.4-99.7) 0.55%

18 1A and (2B or 3B) 51 61 15 12894 45.5 (36.3-54.8) 99.9 (99.8-99.9) 77.3 (67.2-87.4) 99.5 (99.4-99.6) 0.51%

19 1B and (2A or 3B) 54 58 14 12895 48.2 (39.0-57.5) 99.9 (99.8-99.9) 79.4 (69.8-89.0) 99.6 (99.4-99.7) 0.52%

20 1B and (2B or 3B) 51 61 14 12895 45.5 (36.3-54.8) 99.9 (99.8-99.9) 78.5 (68.5-88.5) 99.5 (99.4-99.6) 0.50%

21 1A and (2A or (3A and 4)) 60 52 16 12893 53.6 (44.3-62.8) 99.9 (99.8-99.9) 78.9 (69.8-88.1) 99.6 (99.5-99.7) 0.58%

22 1A and (2B or (3A and 4)) 59 53 14 12895 52.7 (43.4-61.9) 99.9 (99.8-99.9) 80.8 (71.8-89.9) 99.6 (99.5-99.7) 0.56%

23 1B and (2A or (3A and 4)) 60 52 13 12896 53.6 (44.3-62.8) 99.9 (99.8-100.0) 82.2 (73.4-91.0) 99.6 (99.5-99.7) 0.56%

24 1B and (2B or (3A and 4)) 59 53 13 12896 52.7 (43.4-61.9) 99.9 (99.8-100.0) 81.9 (73.1-90.8) 99.6 (99.5-99.7) 0.55%

25 1A and (2A or (3B and 4)) 54 58 11 12898 48.2 (39.0-57.5) 99.9 (99.9-100.0) 83.1 (74.0-92.2) 99.6 (99.4-99.7) 0.50%

26 1A and (2B or (3B and 4)) 51 61 9 12900 45.5 (36.3-54.8) 99.9 (99.9-100.0) 85.0 (76.0-94.0) 99.5 (99.4-99.6) 0.46%

27 1B and (2A or (3B and 4)) 54 58 9 12900 48.2 (39.0-57.5) 99.9 (99.9-100.0) 85.7 (77.1-94.4) 99.6 (99.4-99.7) 0.48%

28 1B and (2B or (3B and 4)) 51 61 9 12900 45.5 (36.3-54.8) 99.9 (99.9-100.0) 85.0 (76.0-94.0) 99.5 (99.4-99.6) 0.46%

29 2A or (1A and 3A and 4) 62 50 26 12883 55.4 (46.2-64.6) 99.8 (99.7-99.9) 70.5 (60.9-80.0) 99.6 (99.5-99.7) 0.68%

30 2B or (1A and 3A and 4) 61 51 24 12885 54.5 (45.2-63.7) 99.8 (99.7-99.9) 71.8 (62.2-81.3) 99.6 (99.5-99.7) 0.65%

31 2A or (1B and 3A and 4) 62 50 25 12884 55.4 (46.2-64.6) 99.8 (99.7-99.9) 71.3 (61.8-80.8) 99.6 (99.5-99.7) 0.67%

32 2B or (1B and 3A and 4) 61 51 23 12886 54.5 (45.2-63.7) 99.8 (99.7-99.9) 72.6 (63.1-82.2) 99.6 (99.5-99.7) 0.65%

____________________________________________________________________________________________________________________________

The criteria used in the definition (1A, 1B, 2A, 2B, 3A, 3B and 4) are given in Table 2 in the text. The prevalence of the patients with RA (N=112) was 0.85% in the population of 13,021 patients.

**Appendix 4**

Sensitivity analysis to evaluate the effect of potential misclassification of patients with rheumatoid arthritis (RA) who did not meet “Definitions of possible cases of RA” (Table 1) as patients having no RA on the prevalence of definition–positive patients and the adjusted prevalence

In the current validation study, from 13,022 patients, we selected 334 possible cases of rheumatoid arthritis (RA) who met the definition of “possible cases of RA” in Table 1 and 12,688 subjects who did not meet the definitions were considered to have no RA. However, it is possible that some of 12,688 subjects who did not meet the definition of “possible cases of RA” had RA. To evaluate the effect of such misclassification, we assumed that the proportion of patients with true RA who met the definitions of “possible cases of RA” and included in 334 possible cases was F and the remaining subjects (with the proportion of 1-F) were misclassified as a patient having no RA. The scenario is analyzed using a 2X2 table in Table S4a1 for the validation study.

|  |  | Reference Standard | | Total |
| --- | --- | --- | --- | --- |
|  |  | Yes | No |  |
| Definition | Yes | a_0_ | b_0_ | a_0_+ b_0_ |
|  | No | c_0_^*^ | d_0_^*^ | c_0_^*^+ d_0_^*^ |
|  | Total | a_0_+ c_0_^*^ | b_0_+ d_0_^*^ | N_0_ (= a_0_+ b_0_+ c_0_^*^+ d_0_^*^) |

Table S4a1: 2X2 table showing the observed number of patients in the validation study where some of c_0_^*^ and d_0_^*^ cases are potentially misclassified

In Table S4a1, a_0_, b_0_, c_0_^*^ and d_0_^*^ indicate the number of true positive (TP), false positive (FP), false negative (FN) and true negative (TN) cases, respectively. When Definition 23 in Table 4 in the text is used, a_0_=53, b_0_=13, c_0_^*^=20, d_0_^*^=12,896, and N_0_=12,982 (Note that one subject whose medical chart was unavailable and 39 subjects who were judged to have RA but the medical care for RA was given in a different hospital were removed from the study population).

The sensitivity (SE^*^) and specificity (SP^*^) in the validation study are estimated, respectively, as:

${SE}^{*}=\frac{a_{0}}{a_{0}+{c_{0}}^{*}}$ (1)

${SP}^{*}=\frac{{d_{0}}^{*}}{b_{0}+{d_{0}}^{*}}$ (2)

Table S4a2 below shows the true number of TP, FN, FP, and TN cases in the validation study. The number of patients for TP (a_0_) and FP (b_0_) in Table S4a1 is the same as those in Table S4a2 because patients who are definition-positive should have the condition code of RA or any DMARD at least in 1 monthly claim during the study period and such a patient meets the first or third criterion of the definition of “possible cases of RA” in Table 1 and should be included in 334 “possible cases of RA”.

|  |  | Reference Standard | | Total |
| --- | --- | --- | --- | --- |
|  |  | Yes | No |  |
| Definition | Yes | a_0_ | b_0_ | a_0_+ b_0_ |
|  | No | c_0_ | d_0_ | c_0_+ d_0_ |
|  | Total | a_0_+ c_0_ | b_0_+ d_0_ | N_0_ (= a_0_+ b_0_+ c_0_+ d_0_) |

Table S4a2: 2X2 table with the number of the true number of TP, FN, FP, and TN patients in the validation study

As the proportion of those with true RA who met the definitions of “possible cases of RA” (a_0_+ c_0_^*^) was F, the following relationship applies:

$F=\frac{a_{0}+{c_{0}}^{*}}{a_{0}+c_{0}}$ (3)

From Equation 3, c_0_ can be given as:

$c_{0}=\frac{\left( 1-F \right)a_{0}+{c_{0}}^{*}}{F}$ (4)

and d_0_ can be expressed as:

$d_{0}={c_{0}}^{*}+{d_{0}}^{*}-c_{0}$ (5)

Using c_0_ in Equation 4 and d_0_ in Equation 5, the true sensitivity (SE) and specificity (SP) are given as:

$\mathrm{SE}=\frac{a_{0}}{a_{0}+c_{0}}$ (6)

$\mathrm{SP}=\frac{d_{0}}{b_{0}+d_{0}}$ (7)

Next, we evaluated the effect of misclassification of true cases of rheumatoid arthritis (RA) as true negative patients in the validation study on the prevalence of the definition-positive patients and the adjusted prevalence of RA in the future study where the validated definition is used. Table S4b below is a 2X2 table for the future study.

|  |  | Reference Standard | | Total |
| --- | --- | --- | --- | --- |
|  |  | Yes | No |  |
| Definition | Yes | a_1_ | b_1_ | a_1_+ b_1_ |
|  | No | c_1_ | d_1_ | c_1_+ d_1_ |
|  | Total | a_1_+ c_1_ | b_1_+ d_1_ | N_1_ (= a_1_+ b_1_+ c_1_+ d_1_) |

Table S4b: 2X2 table with the true values of in the population in the future study to estimate the prevalence

We assume that the prevalence of RA in the future study ((a_1_+ c_1_)/N_1_) differs from the prevalence in the validation study ((a_0_+ c_0_)/N_0_) but SE in Equation 6 and SP in Equation 7 do not differ between two studies. If the true prevalence of RA in the future study is given as P_1_, then a_1_ and b_1_ in Table S4b can be given as:

$a_{1}=P_{1}N_{1}SE$ (8)

$b_{1}=(1-P_{1})N_{1}(1-SP)$ (9)

The prevalence of the definition-positive subjects, P*, is therefore given as:

$P^{*}=\frac{a_{1}+b_{1}}{N_{1}}=P_{1}SE+\left( 1-P_{1} \right)\left( 1-SP \right)$ (10)

When the adjusted number of subjects with RA (B1) is estimated using SE^*^ (Equation 1) and SP^*^ (Equation 2) as B1=(B1*-(1-SP*)$N_{1}$)/(SE*+SP*-1) where the number of definition-positive patients, B1* is described as B1*= a_1_+ b_1,_, the adjusted prevalence, P_adj_ (B1/$N_{1}$) is given as:

$P_{adj}=\frac{\left[ \frac{\left( a_{1}+b_{1} \right)-\left( 1-{SP}^{*} \right)N_{1}}{{SE}^{*}+{SP}^{*}-1} \right]}{N_{1}}=\frac{P_{1}SE+\left( 1-P_{1} \right)\left( 1-SP \right)-\left( 1-{SP}^{*} \right)}{{SE}^{*}+{SP}^{*}-1}$ (11)

We assumed that P_1_=0.56% (the same as in the validation study) or P_1_=1.0% and Definition 23 in Table 4 was used (a_0_=53, b_0_=13, c_0_^*^=20, d_0_^*^=12,896, N_0_=12,982, SE*=0.7260, and SP*=0.9990). F varied between 1 (all patients with true RA were included in 334 “possible cases of RA” in the validation study) and 0.75 (a quarter of true RA patients did not meet the definition of “possible cases of RA” and were misclassified as a patient having no RA in the validation study) and the corresponding P* (Equation 10) and P_adj_ (Equation 11) were calculated for P_1_=0.56% and P_1_=1.0% to produce Figure 2 in the text.
